# Building Resilience against Climate Effects—A Novel Framework to Facilitate Climate Readiness in Public Health Agencies

**DOI:** 10.3390/ijerph110606433

**Published:** 2014-06-20

**Authors:** Gino D. Marinucci, George Luber, Christopher K. Uejio, Shubhayu Saha, Jeremy J. Hess

**Affiliations:** 1 Climate and Health Program, Division of Environmental Hazards and Health Effects, National Center for Environmental Health, Centers for Disease Control and Prevention, Atlanta, GA 30341, USA; E-Mails: ipx1@cdc.gov (G.D.M.); gcl4@cdc.gov (G.L.); cuejio@fsu.edu (C.K.U.); hsf5@cdc.gov (S.S.); 2 Department of Geography, Florida State University, 113 Collegiate Loop, Tallahassee, FL 32306, USA; 3 Department of Environmental Health, Rollins School of Public Health at Emory University, Atlanta, GA 30322, USA; 4 Department of Emergency Medicine, School of Medicine, Emory University, Atlanta, GA 30322, USA

**Keywords:** adaptation, climate change, public health agency, public health, resilience

## Abstract

Climate change is anticipated to have several adverse health impacts. Managing these risks to public health requires an iterative approach. As with many risk management strategies related to climate change, using modeling to project impacts, engaging a wide range of stakeholders, and regularly updating models and risk management plans with new information—hallmarks of adaptive management—are considered central tenets of effective public health adaptation. The Centers for Disease Control and Prevention has developed a framework, entitled Building Resilience Against Climate Effects, or BRACE, to facilitate this process for public health agencies. Its five steps are laid out here. Following the steps laid out in BRACE will enable an agency to use the best available science to project likely climate change health impacts in a given jurisdiction and prioritize interventions. Adopting BRACE will also reinforce public health’s established commitment to evidence-based practice and institutional learning, both of which will be central to successfully engaging the significant new challenges that climate change presents.

## 1. Introduction

Mounting evidence, assembled in numerous national and international-level scientific assessments, strongly indicates that climate change will have broad and significant impacts on infrastructure and a wide range of sectors, including agriculture, transportation, water, and energy management [1,2]. At the nexus of these impacts lie the societal consequences of climate change, well illustrated by the range of challenges to public health. Climate change is likely to have broad public health impacts, from the direct impacts of weather extremes to shifting geographies of infectious diseases and the potential for destabilization of critical societal support systems such as food, energy and transportation. This has led the Director General of the World Health Organization to declare that climate change will be the defining issue for public health in the 21st century [3].

The public health community has identified several potential constraints and barriers to public health adaptation to climate change. These constraints and barriers are not unlike those facing several other sectors [4]. The barriers in public health arise from various sources and include uncertainty about future socioeconomic and climatic conditions as well as a range of financial, institutional, and cognitive limits within public health institutions that can constrain recognition of and action on climate change [5]. To address these barriers, Huang and colleagues have argued that public health needs to put a high priority on research clarifying the potential health impacts of climate change, including scenario-based projections of climate change health impacts; to clarify health co-benefits of potential mitigation strategies; and to evaluate the cost-effectiveness of potential adaptation options [5]. While some of these activities build on established programs and conventional strengths in public health [6], others—particularly scenario-based projections of climate change health impacts—will require the public health community to develop new skills and methodologies, forge new tools, and build new partnerships.

The United States Centers for Disease Control and Prevention (CDC), through the work of its Climate and Health Program, is one of the lead entities supporting state, tribal, local and territorial public health agencies to adapt to climate change in the U.S. [7]. One difficulty that has been identified in readying public health agencies is that climate change is a relatively novel concern and frontline actors in state and local agencies feel unprepared to address the challenges posed by climate change as a result of incomplete knowledge, inadequate staffing, and insufficient training in activities required to facilitate climate change adaptation [8]. Linked with these challenges is the diversity of exposures and health impacts affected by climate change, which includes direct and indirect exposures and associated health impacts [9] that will unfold via place-specific pathways [10]. In many cases our understanding of the systems in which these impacts will unfold is incomplete.

To build climate change adaptation capacity in the public health community, CDC has devised a comprehensive framework for developing local climate change adaptation plans. The Building Resilience Against Climate Effects (BRACE) framework is an iterative approach to adaptively manage the health effects of climate change. The adaptive management approach has shown promise in other sectors, such as the water and natural resources management sectors [11,12] and has been proposed as a potentially useful strategy in public health [11,12,13]. Moreover, BRACE is designed to clarify and assess the most concerning public health risks in a given region, acknowledging the place specificity of many emerging climate change health threats [10]. The CDC’s Climate and Health Program is elaborating BRACE through its implementation in the Climate-Ready States and Cities Initiative cooperative grant program. Through this grant program BRACE is being deployed in 18 public health agencies.

Adaptive management is an iterative, learning-based approach to the design, implementation, and evaluation of interventions in complex, changing systems [14]. Adaptive management explicitly acknowledges that complex systems are incompletely understood, that management interventions can affect system behavior in unexpected ways, and that management strategies need to be regularly updated as system managers and stakeholders learn through interactions with the system and each other [14,15]. Systems well suited to an adaptive management approach are typically incompletely understood and sometimes exhibit unexpected attributes in response to management efforts; ecosystems are an oft-cited example [16]. Adaptive management principles posit that the key to managing these complex, non-linear systems is a learning-based strategy that uses models and emphasizes the need to periodically gather information about their behavior, particularly in response to management actions and shifting stressors over time.

As set forth by the National Research Council in 2004, the adaptive management approach is best applied in settings that exhibit six key elements:

(1) Management objectives that are regularly revisited and revised;
(2) A model of the system(s) being managed;
(3) A range of management choices;
(4) Monitoring and evaluation of outcomes;
(5) Mechanisms for incorporating learning into future decisions; and
(6) A collaborative structure for stakeholder participation and learning [15].

BRACE incorporates adaptive management principles in its recognition that the public health impacts of climate change are complex and that the many systems involved are incompletely understood. BRACE relies explicitly on the use of modeling to project climate change health impacts and regular, iterative reassessment of risks and management priorities as well as integration of new knowledge about how the systems under management are likely to respond to management interventions. In recognition of other barriers and constraints to public health adaptation to climate change identified in the literature, BRACE also requires vulnerability assessment and evidence-based evaluation of potential intervention options. Altogether, these principles are aimed at including recent scholarship on climate change adaptation more generally as well as scholarship on the social determinants of health and integration of learning in public health systems, policy development, and planning [17,18,19,20,21,22,23].

We present the five sequential steps of BRACE and explain how the steps align with the adaptive management principles mentioned above.

## 2. Building Resilience against Climate Effects (BRACE)

BRACE provides for the systematic use of climate projections to inform public health adaptation efforts at a local or regional level [24]. The framework incorporates an assessment of climate change impacts, a vulnerability assessment, the modeling of projected health impacts, an evidence-based evaluation of intervention options, a strategy for implementing interventions, and systematic evaluation of all activities in an iterative framework. Broad stakeholder engagement, adaptive management, and a long range planning frame are key elements.

“*Adaptation*” can be defined as adjustments in ecological, social, or economic systems in response to actual or expected climatic stimuli and their effects or impacts. It refers to changes in processes, practices, and structures to moderate potential damages or to exploit beneficial opportunities associated with climate change [25,26]. In the context of BRACE, *public health adaptation* can be defined as any short- or long-term strategies that can reduce adverse health impacts or enhance resilience in response to observed or expected changes in climate and associated extremes [5]. Interventions are an important component of adaptation for climate change. An *intervention* is defined as a set of actions with a coherent objective to bring about change or produce identifiable outcomes. These actions may include policy, regulatory initiatives, single strategy projects or multi-component programmes. *Public health interventions* are intended to promote or protect health or prevent ill health in communities or populations [27]. In this paper, we regard interventions as actions that will bring about a change or produce an outcome that reduces adverse health impacts. Adaptations other than interventions can be considered strategies that prepare more resilient systems by focusing on building capacity and key capabilities in the public health agency and its partners.

There are five sequential steps in BRACE. Each step aligns with key elements of adaptive management. See Table 1.

### 2.1. The Five Steps of BRACE

**Table 1:** There are five sequential steps in BRACE.

Step No.	BRACE Step Title	Description of Functions	Corresponding Adaptive Management Element
Step 1	**Anticipating Climate Impacts and Assessing Vulnerabilities**	Identify the scope of climate impacts, associated potential health outcomes, and populations and locations vulnerable to these health impacts.	1, 2, 4, 5, 6
Step 2	**Projecting the Disease Burden**	Estimate or quantify the additional burden of health outcomes due to climate change.	1, 2, 4, 5
Step 3	**Assessing Public Health Interventions**	Identify the most suitable health interventions for the health impacts of greatest concern.	1, 3, 4, 5, 6
Step 4	**Developing and Implementing a Climate and Health Adaptation Plan**	Develop a written plan that is regularly updated. Disseminate and oversee the implementation of the plan.	1, 4, 6
Step 5	**Evaluating Impact and Improving Quality of Activities**	Evaluate the process. Determine the value of information attained and activities undertaken.	1, 3, 4, 5, 6

**Figure 1:**
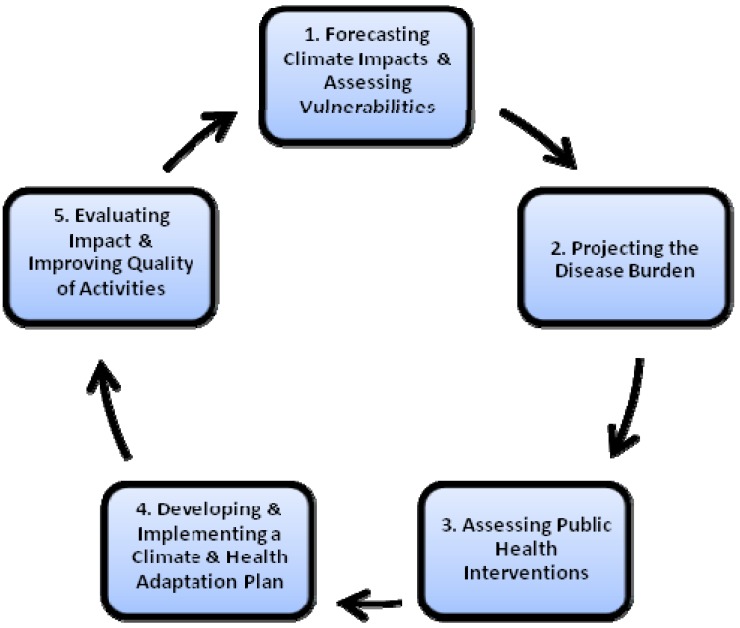
The iterative nature of BRACE.

In line with a key element of adaptive management, BRACE is iterative and enables the incorporation of learning into future decisions. The novel consideration of climate change as a public health concern purports the need for flexibility to insert new or revised information, and improved analyses to assure better decisions can be made. BRACE allows for the inclusion of such information and the re-visit of the Steps to improve decision making and prioritizations throughout the cycle. See Figure 1.

**Figure 2:**
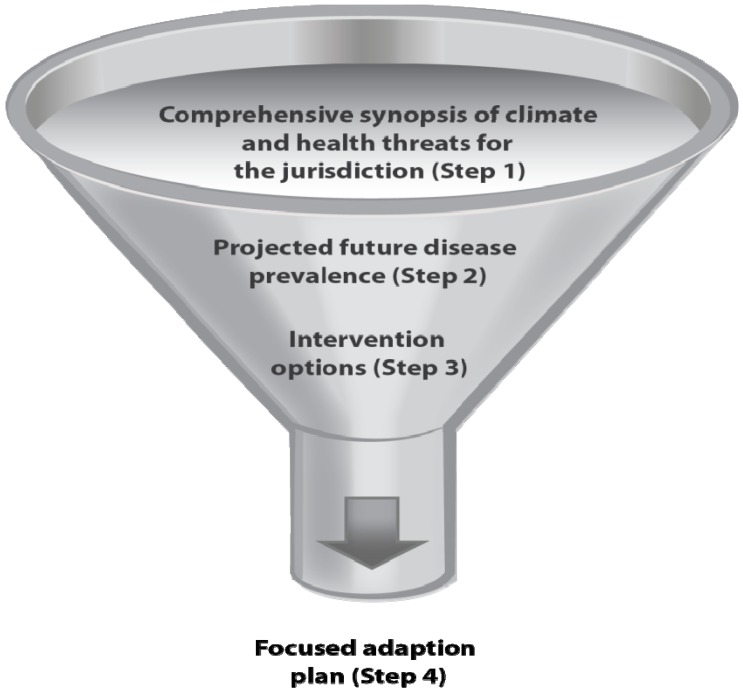
Prioritization of climate and health impacts and suitable interventions.

BRACE enables systematic prioritization of adaptations for resource challenged public health agencies. From the overarching information detailed in Step 1, the hazards of greatest concern can be identified for projection. Step 2 enables further narrowing of scope by providing future disease burden estimates that may help a public health agency choose what issues are of the highest priority for taking action. Additionally, Step 3 allows for decisions about the most effective and locally suitable means for taking action, further prioritizing interventions. These steps culminate in the development and implementation of a locally tailored adaptation plan for public health. See Figure 2.

We describe the steps of BRACE that can be implemented by public health agencies of various sizes and locations and the key considerations for agencies embarking on becoming climate-ready.

### 2.2. Step 1: Anticipating Climate Impacts and Assessing Vulnerabilities

The first step of BRACE involves two inter-related tasks: (1) working with weather, climate variability, and change data sources to identify climate sensitive health outcomes; and (2) identifying vulnerable populations. More specifically, the first task focuses on finding data sources, relating weather to health outcomes and identifying the range of health outcomes that may be affected by climate change and variability within the jurisdiction. Our description includes a summary of key features of climate change projections. By the conclusion of this task a public health agency will have the information to prepare a climate and health profile report (CHPR) that compiles the list of health outcomes of concern for a jurisdiction and details the climate-health exposure pathways.

Then after familiarizing itself with the range of likely climate changes and public health impacts, a public health agency will characterize vulnerability within a jurisdiction. For this second task, we detail multiple methods for describing vulnerability. The CHPR and vulnerability assessment outputs generated in Step 1 serve the purpose of summarizing the range of climate change impacts of concern in the geographic area for policy makers and key stakeholders, and will present information to inspire further investigation and analysis by the public health agency or other interested entities.

#### 2.2.1. Task #1: Working with Weather, Climate Variability, and Climate Change Data Sources to Identify Climate Sensitive Health Outcomes

The public health community will use these data sources to: (a) understand projected climatic changes; (b) identify local weather/climate variability and human health relationships; and (c) obtain climate change projections for quantifying the future disease burden (BRACE Step #2). This can be challenging as public health practitioners are not routinely trained to find, manipulate and interpret this type of information. Working with weather, climate variability, and change information requires specialized technical knowledge. Public health agencies are encouraged to consult with local or regional climatologists who are primary stakeholders for this first task of BRACE.

Organizations skilled in translating climate information (e.g., the National Oceanic and Atmospheric Agency’s Regional Integrated Science and Assessment Centers, State Climatologists) can also help a public health agency to acquire data if pursuing its own analyses (see task #2). Globally, there is significant variance in the development of comparable weather or climate service institutions. The WMO highlights this variance, while demonstrating the importance of such services when available. For example, the WMO highlighted examples of how climate information was improving decision making in Haiti, Mozambique, Fiji, Australia, and China [28]. In addition to these institutions, multiple websites aggregate and direct users to reliable sources of global weather information [29,30,31]. Atmospheric scientists and climatologists have also shared “best practices” for translating and using climate variability and change information to improve decision making [32]. Similar to adaptive management, important practices include mutual education and trust building, joint-knowledge production, sustained collaboration, and sensitivity to organizational and cultural structures that may limit using climate information [33,34,35].

We provide background on climate change projections which are produced by multiple global climate models (GCM) that are driven by future greenhouse gas emissions. GCM are complex computerized simulations of the Earth’s surface, oceans, cryosphere and atmosphere. Models project conditions over relatively large areas (~50–200 km). The clearinghouse for global projections is The World Climate Research Programme Coupled Modeled Intercomparison Project 5 (CMIP5) [36]. Over twenty global climate modeling consortiums share their GCM projections through the data portal. Projecting climate 30 or more years into the future is difficult because uncertainty is created by natural climate variability, incompletely understanding climate feedbacks and climate sensitivities, and societal actions and behaviors [37,38].

Projections are often used to quantify a range of uncertainty surrounding future conditions. Projections are derived from multiple GCM and involve representative concentration pathways (RCP) which cover a broad range of future greenhouse gas (GHG) emissions levels [39,40]. The upper end of the RCP range corresponds to rapid GHG emissions growth (RCP 8.5) while the lower RCP presumes aggressive GHG limits will be enacted (RCP 2.6). The middle pathways suggest that GHG stabilize at different levels (RCP 4.5, 6.0) by the end of the century.

Global data may not be detailed enough for some public health applications. Downscaling translates global climate data into locally relevant climate information. The North American Regional Climate Change Assessment Program and the United States Geological Survey provide state-of-the-art, dynamically downscaled, regional (~50 km) U.S. climate information [41,42]. Comparable initiatives exist for the Mediterranean, South America, East Asia, and Europe (e.g., [43]). We refer readers to in-depth discussions of climate models and downscaling techniques [44,45].

Global climate is projected over different multi-decadal periods from the year 2006 to 2300. Multiple GCM projections are made for each period and RCP since the modelling process is sensitive to initial conditions. Therefore, climate impact studies commonly work with the ensemble or average of GCM runs (1–9 runs) from each model. The ensemble is frequently more accurate than any individual projection [46]. The averaging process tends to cancel random GCM run errors which create an ensemble with the most agreement between runs. Researchers commonly employ two strategies to select GCM projections to use in impact studies. The first strategy presumes GCM have similar levels of skill to reproduce key climatic processes and selects all GCM or a random sample (~10 models) (e.g., [47]). The second strategy selects a subset of GCM that best simulate observed climatic processes. Each global climate modelling group projects future conditions and retrospectively simulates recent climatic conditions (e.g., 1986–2005). For example, researchers concerned with monsoonal precipitation may select GCM with the strongest correspondence between the seasonality and geographic pattern of retrospective simulations and observations (e.g., [48]).

International and national climate change and human health assessments provide an overview of the large range of health outcomes sensitive to climate [9,49,50]. Many climate and health relationships are location-specific. A classic example is an extreme heat event in Oslo, Norway may have the same physical characteristics as an average summer day in Mediterranean Montpellier, France. On average, residents of Montpellier may be more acclimatized (physiological, behavioral, built environment) to extreme heat than people in Oslo [51]. Public health agencies would, therefore, have to develop evidence for local climate and health relationships. Developing these relationships occurs in Step 2 of BRACE by using existing surveillance systems that collect morbidity (e.g., syndromic surveillance) and mortality information. The CDC National Environmental Public Health Tracking Network is an example of a complementary resource that integrates health, exposure, and hazard information into a web-based tool to analyze health impacts associated with environmental exposures that can be used to determine local climate and health relationships. [52,53]. Locally customized efforts are underway in other parts of the world, for example tailored heat response plans are being developed and implemented in India [54] and similarly customized early heat warning systems have been found to be effective in China and Hong Kong [55].

#### 2.2.2. Task #2 Identifying Vulnerable Populations

A vulnerability assessment typically evaluates “the degree to which a system is susceptible to injury, damage, or harm” [56]. Key vulnerability concepts are exposure, sensitivity, and adaptive capacity. Exposure refers to the magnitude, frequency, and duration of an environmental exposure or disease risk. Sensitivity is the ability to withstand the exposure and its aftermath. Adaptive capacity refers to the broad range of responses and adjustments to the potential impact.

There are several methods used to conduct a vulnerability assessment. Vulnerability assessments can take on both qualitative and quantitative components. Quantitative studies tend to assess magnitude and location, to efficiently analyze vulnerability on a large scale. Qualitative studies tend to provide insight of household and individual level pressures from societal factors that are difficult to assess through quantitative methods.

Two approaches to identify vulnerability using Geographic Information Systems include: overlay analyses of risk factors and spatial ecologic studies. Both study designs require locational information (e.g., latitude, longitude) where cases are potentially exposed to hazards. An overlay analysis combines multiple layers of risk factors to spatially define and assess potential vulnerabilities to climate change [57,58,59]. This approach can efficiently analyze vulnerability over large areas, which may be beneficial to populous areas. Publicly available sociodemographic data (e.g., U.S. American Community Survey, tax parcels) provide information on societal vulnerability. Many countries periodically collect comparable census information (e.g., Integrated Public Use Microdata Series) at geopolitical units that can be as small as the neighborhood level [60]. In the absence of existing information, carefully planned household surveys may also provide useful vulnerability assessment information [61,62]. Remotely sensed information (e.g., satellites, Radio Detection and Ranging (RADAR)) can provide neighborhood-level environmental exposure information such as outdoor heat exposure or extreme precipitation rates.

A spatial ecologic study can build upon overlay analysis [63,64,65]. The study determines the most important exposure and vulnerability characteristics associated with a health outcome from observed data. For example, after controlling for population size and other confounders, investigators in Philadelphia, USA, learned that increasing the proportion of vacant households in a local neighborhood by 10%, increased the odds of extreme heat mortality by 40% [64]. Further analysis can be undertaken to assess infrastructure systems that may compound risk, such as distance to hospitals and clinics or utility service areas.

In summary, Step 1 of BRACE draws from multiple elements of adaptive management. For example, management objectives derived from prioritizing climate-sensitive health outcomes and their corresponding planning time frames are chosen in consultation with stakeholders such as local public health agencies, elected officials, planners, academics, community members, and non-governmental organizations (Elements 1, 6). Such consultation can be formal, informal, and structured or unstructured, including meetings, focus groups, open discussion fora, and surveys, among other approaches. Partnering with climatologists will help public health agencies to interpret and work with climate and climate change information (Element 6). Models of climate-sensitive health outcomes can be used to consider both public health risk and societal vulnerability (Element 2).

### 2.3. Step 2: Projecting the Disease Burden

At the conclusion of Step 1, a public health agency has developed a profile of how the climate is changing, the likely effects on health, and the populations and systems most vulnerable to these changes. In Step 2, the agency examines shifting disease burdens more closely. Step 2 can be done qualitatively to yield a general impression of how climate change may affect the risk for certain outcomes, at least capturing general climatic trends and environmental exposures, population vulnerability, and expected human health impacts. However, a quantitative effort, the results of which can be used in comparative health assessments and cost-benefit analyses, is likely to be of greater use to a range of stakeholders if the relevant exposure pathways are adequately understood and if there is sufficient data to drive projections.

**Figure 3:**
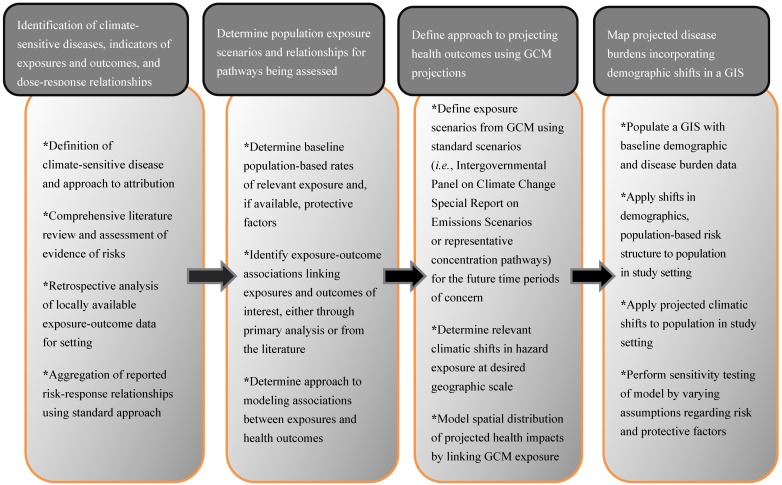
Major steps in projection of climate-associated disease burdens (adapted from [7] with permission from Springer^®^).

From a conceptual standpoint, climate change health impact projections have several major components, as noted in Figure 3.

While a detailed presentation of this process is outside this paper’s scope, the literature on projection of climate change-related health impacts is growing rapidly and there are several different studies that detail relevant methods [66,67,68]. Regardless of the specifics of the chosen approach, the initial step is to define the health outcomes of interest (identified in Step 1 of BRACE) and the climate-health exposure pathway(s) to be assessed. The changing climate presents a novel type of public health challenge in which historic climate and health relationships need to be revisited. Formal quantitative risk assessment using anticipated future disease burden is an unfamiliar exercise for many local public health agencies [8].

There are multiple methods for analyzing and quantifying existing climate and health exposure pathways. We briefly discuss two of the most common statistical methods. Time series and case-cross over studies are similar methods of retrospectively associating weather and climate with morbidity and mortality [69]. These popular study designs have been applied to air pollution, extreme heat, and infectious diseases (e.g., [70,71,72,73]). In a time series, the proportion of cases that temporally coincided with weather events is potentially attributable to weather [74]. Similarly, a case-crossover study compares individual exposures (e.g., ambient pollen levels) before a case-defining event (e.g., an emergency department visit for an asthma exacerbation) to exposures at comparable periods (e.g., ambient pollen levels on the same day as the emergency department visit one week prior or hence) [75].

A population (time series) or individual (case-crossover) is compared against itself to implicitly control for time invariant confounders. Some exposure-response functions are likely to vary by location; if this is anticipated, developing location-specific response functions, if data allow, can help address this heterogeneity. Time series statistics can also be used to evaluate the efficacy of a public health intervention (Adaptive Management Element 4) [76,77].

Several different methods have been used to project disease impacts. While there is no firm consensus regarding the most appropriate approach [78], the most commonly applied technique is the Delta Method, in which changes in the relevant climatic exposure are determined by comparing each GCM’s projected climatic variables (e.g., temperature, humidity, precipitation, *etc.*) in a specified future period with model simulated historical baselines. This standardization process removes model-specific biases common to both the future and simulated historical baseline. Next, the relative difference between the two (the delta) is added to historical baselines. This often provides a more accurate measure of shifting environmental exposure than adjusted GCM variables for projecting future disease burdens. For example, in examining the health impact of increasing temperatures, investigators might examine changes in summertime average maximum temperatures in June, July, and August projected to 2035 and compare these projections with historical data from the specified baseline time period of 1980–2010. The shifted exposure is entered into a function that also contains an exposure-outcome association variable, often a relative risk (typically expressed as a change in relative risk of a specified outcome, e.g., heat-related death, per some fixed interval change in an environmental exposure variable, e.g., 1 °C change in temperature above a particular threshold). The simplest exposure-outcome associations are linear, though any function can be used. If possible, exposure-outcome associations relevant to the population being studied should be used and stratified by relevant demographic information and other factors, but in practice, such detailed information may not be available.

Climate change health impact projections can be data intensive. However, many uncertainties arise, and efforts to increase the precision of projected outcomes may result in unstable estimates due to small sample sizes and other potential biases. In general, the longer the time horizon used in the projection, the greater the uncertainty in the estimates. At a minimum, the data needed to apply the delta method include baseline disease rates (preferably incidence, though prevalence can be used), exposure-outcome associations for the climatic variables being projected, demographic projections for the region being studied, and scenario-based GCM projections of shifts in climatic variables in the study region for the study period. Considerations regarding data sources for projections in Step 2 are listed in Table 2. 

A public health agency should consider whether adaptation should be included in the model. Adaptation activities have the potential to reduce the adverse impacts of climate change [5]. Reasons for including adaptation include general plausibility and methodological consistency specifically if other factors, e.g., demographics shifts that may affect vulnerability are included in the model, in which case it would be reasonable to include adaptation as well. Reasons for leaving out adaptation include a lack of consensus regarding how to model adaptation or an approach in which other factors affecting vulnerability are explicitly left out. Depending on the length of study period (*i.e.*, how far into the future health impacts are projected), projections of likely adaptations—active and passive, planned and unplanned—will be more or less important, as more adaptations will presumably be employed with longer timeframes. There are many different adaptations, some passive (e.g., physiologic adaptation to increasing ambient temperatures) and some active (e.g., usage of mechanical air conditioning) that might be considered.

**Table 2:** Common data sources used in climate change health impact projections (adapted from Hess *et al.* [7] with permission from Springer^®^).

Category of Data Required	Common Data Sources
Baseline rates of disease	Ongoing public health surveillance; published and unpublished regional and national datasets (e.g., National Hospital Ambulatory Medical Care Survey; Healthcare Cost and Utilization Project; Nationwide Emergency Department Sample; Behavioral Risk Factor Surveillance System).
Exposure-outcome associations	Published literature; retrospective analysis of local health outcome datasets merged with local weather and climate data from the National Climatic Data Center or another source; CDC National Environmental Public Health Tracking Network.
Demographic projections	Demographic projections available for the country as a whole from the United States Census; available for individual states via the Federal-state Cooperative for Population Projections.
GCM projections	There are a number of climate models worldwide and certain outputs have been made publicly available; one commonly used source is the Coupled Model Intercomparison Project (CMIP), which issues ensemble model runs for various scenarios (e.g., CMIP3, CMIP5) that are available for download [40,79]; these projections use standard scenarios (e.g., Intergovernmental Panel on Climate Change Special Report on Emissions Scenarios and RCPs) [80].

Including adaptations presumably reduces the likelihood that projected disease burdens will be systematic overestimates, particularly in the long term, and several different methods have been used, but there are no agreed-upon approaches. In some cases, physiologic adaptation to the exposure of concern has been incorporated into exposure-outcome response functions [67]. This approach is viable when there is physiological adaptation, as is the case with ambient temperature changes. In other cases, adaptation has been accounted for by systematically adjusting or discounting estimates of future impacts [81]; this approach is more generic and can be used as a proxy for many different types of adaptation or a mixed combination of adaptation strategies.

While projecting disease burden can be time- and data-intensive, the investment can potentially pay dividends going forward as the models can be used to guide several different types of decisions over time and to engage with various stakeholders to help prioritize risk management decisions. The models can also be further developed and modified as additional information becomes available and thereby feed into adaptive management processes [82].

### 2.4. Step 3: Assessing Public Health Interventions

The focus of Step 3 of BRACE is identifying the most suitable interventions to adapt to the climate change related health threats identified as of greatest concern in Steps 1 and 2. For various clinical and public health interventions, the evidence-based public health (EBPH) approach provides practitioners an opportunity to assess the efficacy of alternative interventions [83]. While the EBPH literature on public health interventions in response to climate change is meager, a recent review lists a wide range of such interventions [84] and outlines the relevant evidence.

The general EBPH approach entails the following steps: (i) assessment of the problem; (ii) a systematic review of the public health literature to identify relevant interventions; and (iii) assessment of the efficacy of interventions [83,85]. In the case of climate change, the problem assessment step includes both assessment of the shifting exposures resulting from climate change and the likely health impacts [86]. Step 3 of BRACE is focused, in particular, on the latter parts of the EBPH process, in which intervention efficacy is examined closely.

While the literature on some potential public health impacts of climate change (e.g., problem assessment) is substantial, there is relatively little information published on specific adaptations and interventions that may avoid or limit these projected impacts. For instance, a recent structured review of population-level interventions to reduce the impacts of extreme heat identified only 14 studies, all of which were cross-sectional or retrospective, and the authors were unable to generate a specific estimate of impacts [87]. As instances of agencies designing and implementing these health interventions across different jurisdictions increase, established guidelines like the Preferred Reporting Items for Systematic Reviews and Meta-analyses [88] could be used to evaluate and compare intervention efficacy.

Even when a range of evidence-based health interventions are available, budget constraints may necessitate comparisons based on cost to help public health agencies prioritize and select across potential alternatives. The comparative cost-effectiveness paradigm [89] provides public health agencies an approach to choose among available intervention and adaptation strategies in this context. By adapting the definition of comparative effectiveness in clinical research developed by the Institute of Medicine [90], this approach requires systematic comparison of available estimates of costs and benefits associated with prevention, diagnosis, treatment, and monitoring of alternative strategies designed to reduce the adverse health impacts from changes in climate-sensitive exposures. Established protocols on conducting comparative effectiveness research using observational data [91] and cost-effectiveness analysis [92] can be adapted to evaluate and identify intervention strategies. While higher levels of evidence are particularly useful for more costly interventions, other forms may be very important in guiding day-to-day decisions that many public health officials encounter in the course of their activities. Some locales may decide it is more appropriate for them to collect their own evidence to guide interventions. Public health practitioners also frequently use anecdotal evidence conveyed through informal professional networks in making ad-hoc decisions when infrequently studied issues arise, such as strategies for promoting the use of cooling centers and making decisions about when to issue heat-health warnings.

Evidence may also not be available for certain potential risks, particularly those associated with cascading system failures like electrical blackouts or sewage treatment failures after extreme precipitation events. In such cases, public health officials may need to access literature outside of public health to identify strategies for promoting resilience across a range of linked systems upon which public health relies.

Overall, while systematic review of the literature and identification of efficacious interventions is of paramount importance, it is also clear that other, less robust forms of evidence will also enter into deliberations regarding the interventions to pursue. As the field matures and various interventions are implemented, public health practitioners may consider prioritizing the reporting of these interventions and their effects using relevant guidelines already in the literature.

In addition to assessing literature and cost-effectiveness for interventions, a public health agency may also consider assessing the suitability of interventions to their political, cultural, and logistical environment. In this instance, a public health agency may consult with a range of stakeholders that will be affected by or play a role in proposed interventions. For example, considering adaptations to extreme heat may require coordination inside public health agencies, with other health partners, local weather forecast offices, non-government organizations and community groups to establish critical temperature thresholds, consider the dissemination of information to vulnerable populations and identify locally appropriate response measures such as the activation of cooling shelters or transportation of immobile seniors to protective environments. Consultation might consider the resources needed and available for the considered interventions, the skill sets and technological assets available or needed, cultural and political palatability and the opportunity cost of taking action. Assessing these elements can provide insight as to the likely acceptance of the action and the likely barriers that will be faced.

Both assessments of intervention efficacy and suitability for the specified setting will feed into the process of identifying interventions that are most likely to be suitable for the jurisdiction. The combined assessments enable the ranking of a set of focused actions that will form the core elements of a locally specific and manageable adaptation plan for public health. Undertaking Step 3 of BRACE employs several elements of adaptive management. This step studies and considers a range of management choices (Element 3) and in its most collaborative form enables extensive opportunities for stakeholder participation and learning (Element 6).

### 2.5. Step 4: Developing and Implementing a Climate and Health Adaptation Plan

The focus of Step 4 of BRACE is synthesizing information generated in the prior steps into a focused climate and health adaptation plan. BRACE emphasizes the need for a unified adaptation plan for the public health sector to foster collaboration across disciplines and interest groups and to align efforts to a common objective of protecting and promoting health. Creating such a plan is not a substitute for integrating public health considerations into broad climate change plans in other sectors. Rather, it should complement and add greater specificity to public health considerations that will be ideally included within a cross-sectoral, jurisdiction wide plan for climate change action.

With respect to adaptation, in practice, stakeholders may use disparate interpretations that reflect different underlying goals and priorities [93]. Explicitly recognizing these differences is important for reconciling goals and developing evaluation metrics for adaptation planning [94]. Frameworks, heuristics, and “best practices” from the climate and global change adaptation and sustainability science literature have been incorporated into BRACE [20,95]. For example, stakeholder engagement, communication, iterative learning, and adaptive management are key principles of both “successful” adaptation frameworks [25,94] and BRACE, and are reflected in BRACE guidance for climate and health adaptation plans.

A climate and health adaptation plan might aim to coordinate, highlight, and, potentially instigate a series of activities aimed at preventing, or at least reducing, the anticipated impacts of climate change in the area. Activities in the plan may be comprised of new, enhanced, or established programs and activities and the plan may outline or reinforce how these activities will be implemented and their success measured. The sponsoring public health agency may also consider including a means to engage with other key sectors that have responsibility for policies, programs, or oversight that will ultimately affect public health outcomes. For example, developing memorandums of understanding that foster greater exchange of information, skills and resources between a public health agency and agencies responsible for housing, planning, transportation and water quality, might spur earlier input into development plans that can further optimize public health benefits or mitigate unintended harm. A public health agency might consider a means for the adaptation plan to be periodically updated, and ensuring public accessibility of the plan.

In preparing an adaptation plan, a public health agency should aim to be comprehensive, considering options for action that cut across all of the essential public health functions from surveillance to regulation to outreach and education [6]. However, a plan must also be balanced with the feasibility considerations detailed in Step 3 to increase the probability of plan uptake and implementation. A plan should identify how stakeholders can integrate adaptations into their existing programs and should detail how activities will be evaluated. The scope of the plan should be intersectoral while maintaining a focus on public health outcomes. The planning horizon for the activities contained within the plan should span several years.

In preparing a climate and health adaptation plan, a public health agency can adapt the key elements and structure found in many jurisdiction wide, multisectoral climate change adaptation plans. A typical Climate Change Adaptation Plan has the following elements:

- Community profile which includes background information
- Most appropriate” regional/municipal climate change scenario
- Scoped local climate change impacts
- Prioritized consequences/prospects of risks and opportunities
- Maps showing priorities
- Adaptation planning principles
- Table of recommended adaptation policies and actions indicating priority, lead responsibility and fit with existing program (if applicable)
- Action plan for tasks to be accomplished in the community
- Community engagement process
- List of key stakeholders
- Inventory of risks and opportunities
- Inventory of consequences and prospects
- Gap analysis of programs useful for adaptation actions [96]

Public health agencies should anticipate that climate and health adaptation plans will be used as internal and external communication documents. Therefore plans should clearly outline the resources required to undertake the series of activities, detail how established programs may need to be modified to cater for changing risk factors and environmental conditions, and identify the parties responsible for executing the activities. As discussed earlier, some responsibilities may lie in partnerships with other agencies. The role of these agencies, the mechanism for their engagement and the means for coordinating should be included in the plan, and the nature of the collaboration must be well described. The public health agency should include clear language expressing its commitment to communicate its findings and updates to stakeholders throughout the course of the plans implementation. For stakeholders outside of the public health agency, the plan will provide a vision for protecting health against the effects of climate change in the jurisdiction and can be used to educate prospective partners on ways they can contribute to the strategy. Finally, the plan should explicitly detail future review and periodic revisions of the plan to ensure that the projected health threat and adaptation strategies do, in fact, represent the most appropriate path forward for protecting the public’s health.

To initiate implementation of the plan the public health agency must assure its comprehensive dissemination among a wide range of stakeholders that may play a role in implementing the stated activities.

The process of developing and implementing a climate and health adaptation plan crosses all of the elements of adaptive management. In particular, a plan is a means for defining the management objectives (Element 1) of the strategy, the prioritized interventions and adaptations (Element 3), measures for monitoring and evaluating progress (Element 4), quality improvement processes (Element 5) and the role of stakeholders (Element 6).

### 2.6. Step 5: Evaluating Impact and Improving Quality of Activities

Implementing BRACE is an iterative process. Climate change considerations are relatively new for the public health community, therefore, gathering information on the processes used to address the health effects of climate change and the potential outcomes and impacts of those processes are critical. It is important to note that, while evaluation is positioned in Step 5 to better accommodate discussion and communication, in reality, monitoring and evaluation of processes, outcomes and key indicators are central considerations throughout the entire process. The value of explicitly making processes iterative is that management decisions can be revisited with new information, not only general knowledge about the threats being managed, but also information gleaned from experience since the process began. This ability to continually improve interventions is a fundamental adaptive management tenet.

At any point in the implementation of BRACE, process evaluation measures can help to validate methods employed and to reveal flaws in the plan. In addition to assessing the execution of key methods, process metrics can help to determine if the most appropriate stakeholders have been engaged and if the stakeholders’ engagement added critical input. Evaluation can also identify the outcomes that resulted from the combined series of activities, for example, improved capacity to develop models, stronger partnerships with key stakeholders, identification of additional surveillance needs, and awareness of synergies across programs. Ultimately, the evaluation step can help to determine the impact of implementing BRACE by determining the extent to which the interventions improved public health outcomes. Evaluating impact is influenced by the quality of assessment performed in Steps 1 and 2 of BRACE. Rigorously evaluating public health intervention requires baseline climate and health relationships (Step 1) [97]. Similarly, adaptations can be evaluated against counterfactuals (Step 2) that estimate climate change attributable disease burden in the absence of adaptation.

While a comprehensive discussion of evaluation methodology is outside the scope of this paper, it is expected that monitoring and evaluation methods will be used in the implementation of BRACE to gauge progress. While each agency will have different evaluation resources, the agency should be able to answer some basic questions after its evaluation activities:

- Does the public health agency have a reasonable estimate of the health impacts of climate change in its jurisdiction?
- Has the process allowed the public health agency to prioritize health impacts of greatest concern and the most suitable interventions?
- Has the public health agency prepared an adaptation plan for the public health sector within the jurisdiction?
- Are climate change considerations accommodated in public health planning and implementation activities?
- Are public health considerations accommodated in climate change planning and implementation activities?
- Are indicators in place that will evaluate the interventions implemented as a result of utilizing BRACE?
- How can the process be improved in the next iteration?
- What are the agency’s top learning priorities in the next iteration of BRACE?

The long-standing tradition of institutional learning from responses to novel threats places the public health community in a strong position to overcome many of the potential constraints and barriers to climate change adaptation identified in the introduction [5]. Public health agencies can accelerate the learning needed by programs to more effectively address the health impacts of climate change by employing rigorous monitoring and evaluation processes. To achieve this public health agencies may consider how they can sustain their commitment to learning, the need for new skills and consideration of longer planning horizons [5].

## 3. Key Considerations for Implementing BRACE

There are some key points to consider in the implementation of BRACE. First, eliciting stakeholder viewpoints and perspectives can add significant value to the overall process. While stakeholder engagement is critical in each step, BRACE is an adaptive management framework and therefore calls for periodic revisions to the stakeholder network in order to better align with the specific goals of each step. For example, in Steps 1 and 2, where the emphasis lies on the integration of climate change scenarios into the projection of health effects, a public health agency may gain significantly from engagement with the climate science community, as the expertise of generating and using climate projections may not exist within the agency. In Step 3, which focuses on the assessment and identification of appropriate public health actions and interventions, it would be appropriate to solicit input from the larger public health practitioner and health care provider communities and other non-health agencies that may share co-benefits.

A second point to consider while implementing BRACE is the temporal scope for assessing changing climatic conditions and for choosing adaptations or key interventions. Factors such as quality of data and certainty must be balanced against considerations of a public health agency’s level of readiness and the future capacity needed to assure that the public health system will be able to cope with novel threats or significant increases in high risk exposures. A public health agency may anticipate needing to deploy certain interventions, which provide insight into key timeframes for planning and assessment. For example, a public health agency that anticipates that extreme heat events are of concern may target interventions linked to city planning. While the public health community operates on short-term horizons, the planning community deals with long term issues, mediating the use of space and shaping future development [98]. Typically, city or regionals plans are prepared with a 20–25 year planning horizon [99,100,101,102,103]. Planners need to have the understanding to integrate future health matters into their day-to-day considerations [98]. In order to provide scientifically credible inputs into these planning processes, public health agencies can benefit from modeling disease outcomes that may be influenced by environmental conditions in similar time periods.

A public health agency anticipating the effects of sea level rise on drinking water and wastewater infrastructure may consider a longer planning horizon. For example, the American Water Works Association estimates that drinking water and waste water pipes laid in post-World War II can be expected to last about 75 years. A public health agency that seeks to assess threats associated with sea level rise may need to focus on projected exposures and risk factors closer to a 75-year timeframe, to accommodate wastewater and drinking water infrastructure planning considerations [104].

The third key point relates to how a public health agency prioritizes the health impacts to be addressed and the interventions to be employed. Changing climatic conditions will directly or indirectly impact many health outcomes. This broad range of health outcomes, combined with limited experience with using and interpreting climate change projections, can be disconcerting and overwhelming for a public health manager considering a course of action to manage these threats [8,105]. Shrinking resources and competing demands on public health agencies further compound the challenges associated with taking action [106,107]. BRACE provides a system to manage the information provided in climate change projections, triage the health outcomes of concern, and prioritize the most suitable interventions for the jurisdiction.

## 4. Summary and Conclusions

Climate change is an evolving concern. For public health agencies, climate change presents a number of adaptation challenges, not least of which is the incompletely understood nature of the impacts and the ecological systems in which these impacts will unfold. Managing the public health risks associated with climate change requires an iterative framework consistent with the principles of adaptive management. BRACE incorporates the features of adaptive management into a stepwise process tailored for public health agencies. BRACE is currently in a proof-of-concept phase and is laid out here for dissemination beyond the locales in which it is being piloted. Following the steps laid out in BRACE should enable a public health agency to use the best available science to assess current and future climatic conditions and prioritize the health outcomes and interventions that are most important and suitable for the jurisdiction.

BRACE implementation thus serves as an opportunity for learning in public health. We support efforts of public health agencies implementing BRACE, to evaluate how the framework helps them negotiate various barriers and limits to climate change adaptation, and where it can be revised. BRACE incorporates “best practices” from the broader climate change adaptation literature while providing flexibility for public health agencies to develop innovative and rigorous adaptation case studies. Similarly, these agencies will evaluate how well adaptive management facilitates working with complex systems that they attempt to influence. BRACE pragmatically recognizes that public health and climate change are two of many considerations for making adaptation policy decisions.

Preliminary feedback from participants in CDC’s Climate-Ready States and Cities Initiative, points to the complexity in accessing, interpreting and using climate model projections for empirically projecting the disease burden. This necessitates the use of sophisticated modeling expertise that may prove challenging for public health agencies. The level of specificity needed for disease projections will become more apparent as this information informs public health adaptations going forward. How successfully public health agencies navigate this challenge depends on the collaborations built with agencies involved in developing climate projections.

Acknowledging that BRACE may need to be refined as evidence regarding its implementation accumulates, we feel it is important to highlight that adopting BRACE serves as a ratification of public health’s established commitment to evidence-based practice and institutional learning. Both will be paramount as public health wrestles with the significant new challenges that climate change presents. The implementation of BRACE across a variety of settings serves as an opportunity for evaluation of the framework’s utility and, indirectly, of the utility of the principles of adaptive management upon which it is built. In this way, BRACE may serve to advance the science related to climate change adaptation not only within public health but more generally.
